# AI Denoising Significantly Enhances Image Quality and Diagnostic Confidence in Interventional Cone-Beam Computed Tomography

**DOI:** 10.3390/tomography8020075

**Published:** 2022-04-01

**Authors:** Andreas S. Brendlin, Arne Estler, David Plajer, Adrian Lutz, Gerd Grözinger, Malte N. Bongers, Ilias Tsiflikas, Saif Afat, Christoph P. Artzner

**Affiliations:** Department of Diagnostic and Interventional Radiology, Eberhard-Karls University, D-72076 Tuebingen, Germany; arne.estler@med.uni-tuebingen.de (A.E.); david.plajer@med.uni-tuebingen.de (D.P.); adrian.lutz@lutz-ing.de (A.L.); gerd.groezinger@med.uni-tuebingen.de (G.G.); malte.bongers@med.uni-tuebingen.de (M.N.B.); ilias.tsiflikas@med.uni-tuebingen.de (I.T.); saif.afat@med.uni-tuebingen.de (S.A.); christoph.artzner@med.uni-tuebingen.de (C.P.A.)

**Keywords:** cone beam computed tomography, AI (artificial intelligence), image quality enhancement

## Abstract

(1) To investigate whether interventional cone-beam computed tomography (cbCT) could benefit from AI denoising, particularly with respect to patient body mass index (BMI); (2) From 1 January 2016 to 1 January 2022, 100 patients with liver-directed interventions and peri-procedural cbCT were included. The unenhanced mask run and the contrast-enhanced fill run of the cbCT were reconstructed using weighted filtered back projection. Additionally, each dataset was post-processed using a novel denoising software solution. Place-consistent regions of interest measured signal-to-noise ratio (SNR) per dataset. Corrected mixed-effects analysis with BMI subgroup analyses compared objective image quality. Multiple linear regression measured the contribution of “Radiation Dose”, “Body-Mass-Index”, and “Mode” to SNR. Two radiologists independently rated diagnostic confidence. Inter-rater agreement was measured using Spearman correlation (r); (3) SNR was significantly higher in the denoised datasets than in the regular datasets (*p* < 0.001). Furthermore, BMI subgroup analysis showed significant SNR deteriorations in the regular datasets for higher patient BMI (*p* < 0.001), but stable results for denoising (*p* > 0.999). In regression, only denoising contributed positively towards SNR (0.6191; 95%CI 0.6096 to 0.6286; *p* < 0.001). The denoised datasets received overall significantly higher diagnostic confidence grades (*p* = 0.010), with good inter-rater agreement (r ≥ 0.795, *p* < 0.001). In a subgroup analysis, diagnostic confidence deteriorated significantly for higher patient BMI (*p* < 0.001) in the regular datasets but was stable in the denoised datasets (*p* ≥ 0.103).; (4) AI denoising can significantly enhance image quality in interventional cone-beam CT and effectively mitigate diagnostic confidence deterioration for rising patient BMI.

## 1. Introduction

Despite advances in classical oncological therapy, primary and secondary hepatic malignancies are associated with poor outcomes and are generally considered the limiting factor to overall survival [1]. Therefore, many prominent guidelines define radical resection as the curative treatment of choice for hepatic malignancies, regardless of their origin [2,3,4]. Unfortunately, numerous patients with malignant liver lesions are disqualified from curative resection due to the number or location of the lesions, their proximity to vascular structures, or insufficient parenchyma reserve [5]. However, recent review articles have pointed at the possible benefit of liver-directed interventions to decrease tumor burden and, at best, to re-allow surgical resection [6]. Prominent interventional radiological procedures include transarterial chemoembolization (TACE) with or without drug-eluting beads and selective internal radiation therapy (SIRT) with yttrium-90 spheres [7,8]. In the last decade, such interventional procedures have benefitted from improved visualization due to cone-beam computed tomography, a technique enabling cross-sectional images via rotating the c-arm equipped with a flat-panel detector around the patient [9]. During the procedure, parenchyma blood volume (PBV) overlay maps can be used to localize the interventional target lesion [10]. Such PBV overlay maps are typically acquired by subtraction of cone-beam computed tomography (CT) via two C-arm rotations, one unenhanced “mask” run, and one contrast-enhanced “fill” run [11]. Although modern interventional radiology suites are usually equipped with automatic radiation dose adaption to preserve signal yield, recent studies have pointed out image quality deterioration for higher patient body mass index (BMI) [12,13]. In conventional CT imaging, recent AI postprocessing solutions show promising potential for significant image quality enhancement [14]. However, like all novel techniques, AI-based postprocessing is associated with typical pitfalls, such as loss of information and spatial resolution deterioration via increased blurring [15]. Therefore, review articles covering this topic have consecutively suggested investigating AI solutions strictly on a use-case level [16]. And although several prior studies have evaluated the impact of AI postprocessing on image quality in other modalities, investigating the effects of denoising on interventional cone-beam CT regarding patient BMI has, to the best of our knowledge, not yet been attempted [17,18,19]. Therefore, we aimed to investigate if interventional cone-beam CT could benefit from AI denoising, especially when performing subgroup analyses for patient BMI. We hypothesize that denoising may help mitigate image quality deterioration for rising patient BMI in this setting.

## 2. Materials and Methods

### 2.1. Study Population and Radiation Dose

An a priori power analysis using the software solution G*Power (ver. 3.1.9.7, Franz Faul, University of Kiel, Germany) determined the necessary sample size (*f* = 1.18, α = 0.05, 1-β = 0.95) to be 100 patients [20]. The local ethics committee approved the analyzing of our center’s in-patients for eligibility from 1 January 2016 to 1 January 2022 with a waiver for the need for informed consent (#167/2020BO2). Initially, we collected clinical indications, type of interventional procedure, and whether patients had received dual-phase C-arm CT during the procedures. If a patient had received multiple interventional procedures in the given timeframe, we only included the most recent and removed the others (“duplicates”). Further exclusion criteria were: no oncological indication, no SIRT/TACE, and only single-phase c-arm CT during the procedure. From the included 100 patients, we collected age, sex, height, weight, and their body mass index (BMI in kg/m^2^) at the procedure time from their clinical reports. Furthermore, the patients were assigned BMI subgroups (BMI ≤ 24 = normal weight, BMI 25–28 = pre-obesity, BMI ≥ 29 = obesity) according to appropriate reference data and rounded to the nearest whole number to facilitate comprehensive subgroup testing [21]. From the dose reports of the procedure, we collected the dose-area-product (in mGy*cm^2^) of the corresponding series.

### 2.2. Image Acquisition, Reconstruction, and Postprocessing

All images were acquired in interventions performed on the same multiaxis robotic angiographic C-arm suite (Artis Zeego Q, VE40 A, Siemens Healthcare, Forchheim, Germany). Parenchyma blood volume (PBV) maps were acquired by an unenhanced rotation (“mask”) and a contrast-enhanced return-rotation (“fill”). The acquisition time per rotation was 4 s and the total examination time was 16 s. The X-ray tube was set to 90 kilovolts and covered a total angle of 200° (0.8° per frame, 248 frames). The matrix size was 616 × 480 pixels, the flat-panel pixel size was 616 μm, and the mean radiation dose exposure was 0.36 μGy per frame. For the fill run, a contrast media dilution of 30 mL (7.5 mL: Ultravist 370, Ultravist 370, Bayer Schering, Zurich City, Zurich, Switzerland + 22.5 mL: saline solution) was injected through an antecubital vein cannula using a dual-head power injector (Accutron-HP-D, Medtron, Saarbrücken, Germany) at a flow rate of 3 mL/s. The raw image data was automatically sent to a commercially available offline workstation (syngo XWP, Siemens Healthcare). Subsequently, automatic reconstruction of CT-like axial images with a slice thickness and increment of 1 mm ensued, using a weighted filtered back-projection algorithm with a median filter. Additionally, all reconstructions were post-processed using a novel AI denoising algorithm (PixelShine^®^, AlgoMedica, Sunnyvale, CA, USA). Subtractions for vascular input function calculation and parenchymal blood volume (PBV) overlay maps were created in MatLab (Ver. R2021a, The MathWorks, Natick, MA, USA).

### 2.3. Objective Image Quality

For objective image quality analysis, all corresponding series (regular: mask & fill, denoising: mask & fill) per patient were loaded into the open-source ImageJ distribution FIJI (ver. 1.53k, Wayne Rasband, National Institutes of Health-NIH, Bethesda, MD, USA) [22]. The non-denoised fill series were used to draw a total of 30 regions of interest (ROI) with a diameter ≥ 1 cm^2^ into homogenous areas of liver parenchyma outside of the vessels, bile ducts, and cancerous lesions. The program then conveyed those ROIs into each loaded series per patient and performed consistent measurements of mean CT numbers in Hounsfield Units (HU) and their standard deviation (SD). The SD of HU was defined as image noise. A signal-to-noise ratio (HU/SD) per ROI was computed as a comprehensive measure for objective image quality.

### 2.4. Diagnostic Confidence

The patient datasets were anonymized and randomized by a group member otherwise not associated with diagnostic confidence analysis. Two radiologists with 5 and 10 years of experience independently rated diagnostic confidence on a 5-point Likert scale (1 = poor, 2 = subpar, 3 = fair, 4 = good, 5 = excellent). All ratings per patient and dataset (regular/denoising) were pooled for an overall grade.

### 2.5. Statistical Analysis

Statistical analysis and illustration were performed using GraphPad Prism version 9.3 for Windows (GraphPad Software, San Diego, CA, USA). Data distribution was tested using the Shapiro–Wilk test. Normally distributed variables were expressed as mean ± standard deviation and non-normally distributed variables as median and interquartile range (IQR). Data analysis ensued using a mixed-effects model with Greenhouse–Geisser correction in case of violation of sphericity. Post-hoc tests were performed for BMI subgroups. Two-stage step-up correction after Benjamini, Krieger, and Yekutieli was utilized to counteract type 1 error increase in multiple comparisons. An adjusted *p*-value ≤ 0.05 indicated statistical significance. Multiple linear regression was utilized to investigate the effect of the variables “BMI” (normal weight/pre-obesity/obesity, reference category: normal weight), “Radiation Exposure” (dose-area-product in mGy*cm^2^), and “Mode” (regular/denoising, reference category: regular) on the signal-to-noise ratio. The utility and goodness-of-fit of the model were measured using variance (ANOVA), adjusted R^2^, and the standard deviation of the residuals (Sy.x). R^2^ values of ≤0.13 were considered indicative for poor, 0.13–0.26 for moderate, and ≥0.26 for high goodness-of-fit [23]. We computed Spearman’s correlation coefficient (*r*) to measure inter-rater-agreement for the diagnostic confidence ratings. We defined r-values of 0–0.5 as indicative for poor, 0.51–0.74 for moderate, 0.75–0.9 for good, and 0.91–1.00 for excellent agreement.

## 3. Results

### 3.1. Study Population and Radiation Dose

**Figure 1:**
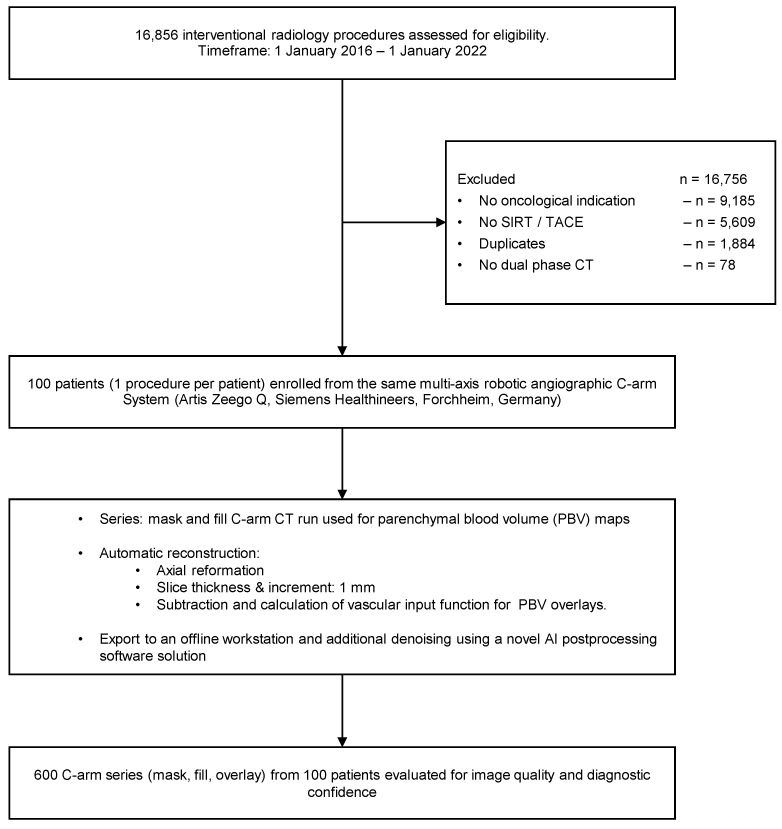
Patient enrollment and study workflow.

A total of 16,856 procedures were evaluated for eligibility, 16,756 procedures were excluded, and 100 procedures were included. From these 100 procedures, 600 datasets were generated through reconstruction and postprocessing to investigate image quality and diagnostic confidence further. Figure 1 visualizes patient enrollment and the study workflow.

Of the 100 included patients (25 female/75 male, mean age 68 ± 11 years, mean BMI 27 ± 4), 50 patients received selective internal radiation therapy (SIRT), and 50 patients received transarterial chemoembolization (TACE). A total of 60 patients had hepatocellular carcinoma (HCC), 17 patients had metastasized uveal melanoma (mUM), 10 patients had metastasized neuroendocrine tumor (NET), eight patients had metastasized cholangiocellular carcinoma (CCC), and five patients had metastasized colorectal carcinoma (CRC). See Table 1 for further details.

**Figure 2:**
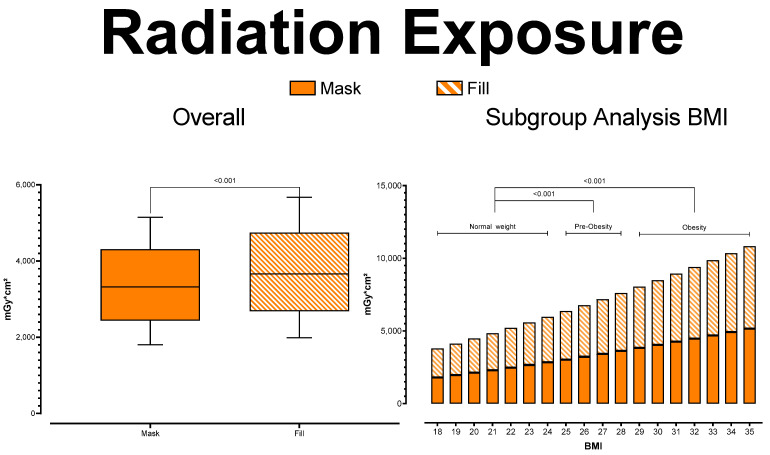
Radiation Exposure.

Mean radiation exposure was 3363.01 ± 690.79 mGy*cm^2^ for the mask series (normal weight: 2502.6 ± 308.98 mGy*cm^2^; pre-obesity: 3342.29 ± 223.13 mGy*cm^2^; obesity: 4140.46 ± 364.03 mGY*cm^2^) and 3705.20 ± 761.08 mGy*cm^2^ for the fill series (normal weight: 2757.25 ± 340.41 mGy*cm^2^; pre-obesity: 3682.38 ± 245.83 mGy*cm^2^; obesity: 4561.76 ± 401.07 mGY*cm^2^). As expected, the post-hoc subgroup analysis showed radiation exposure to rise significantly with rising patient BMI in both series (each *p* < 0.001). See Figure 2 for further details.

### 3.2. Objective Image Quality Analysis

**Figure 3:**
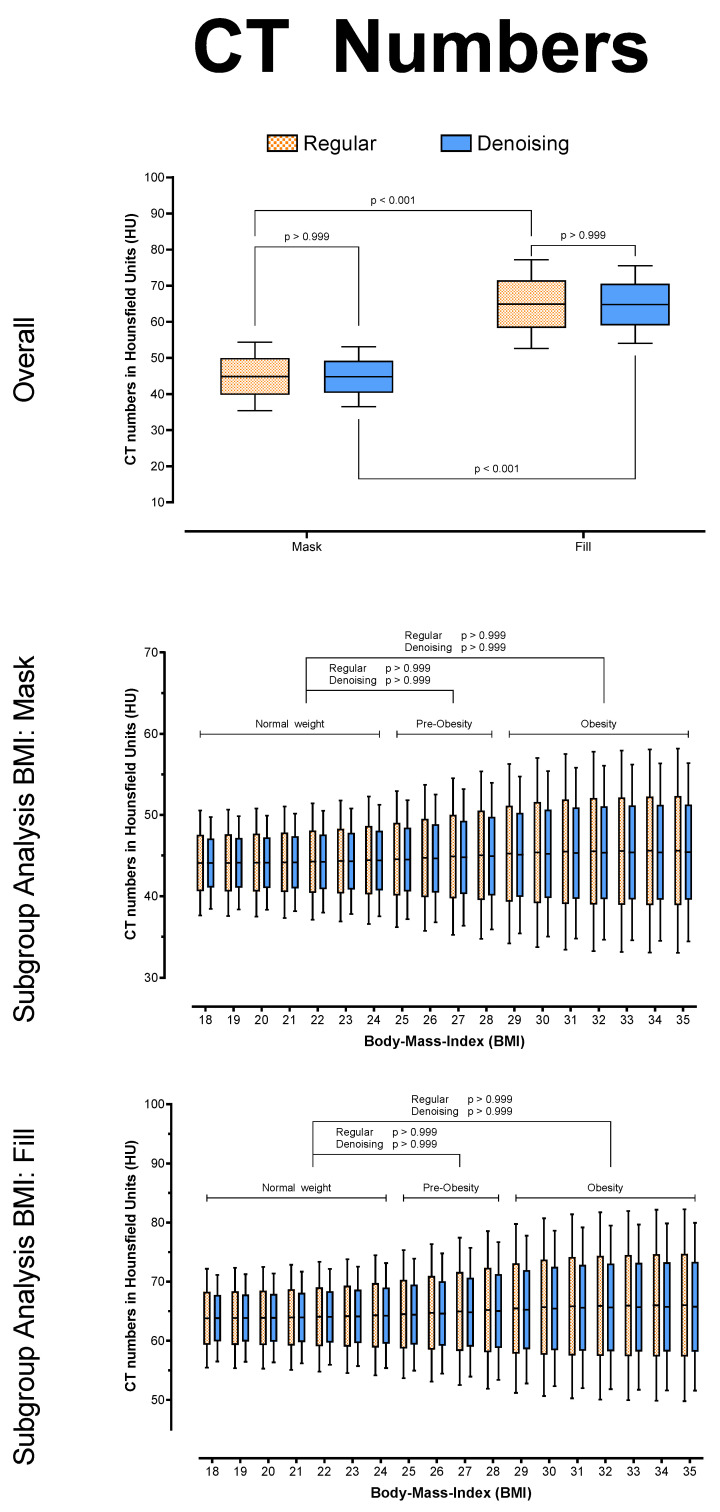
CT numbers in HU, overall and posthoc subgroup analysis for patient BMI.

**Figure 4:**
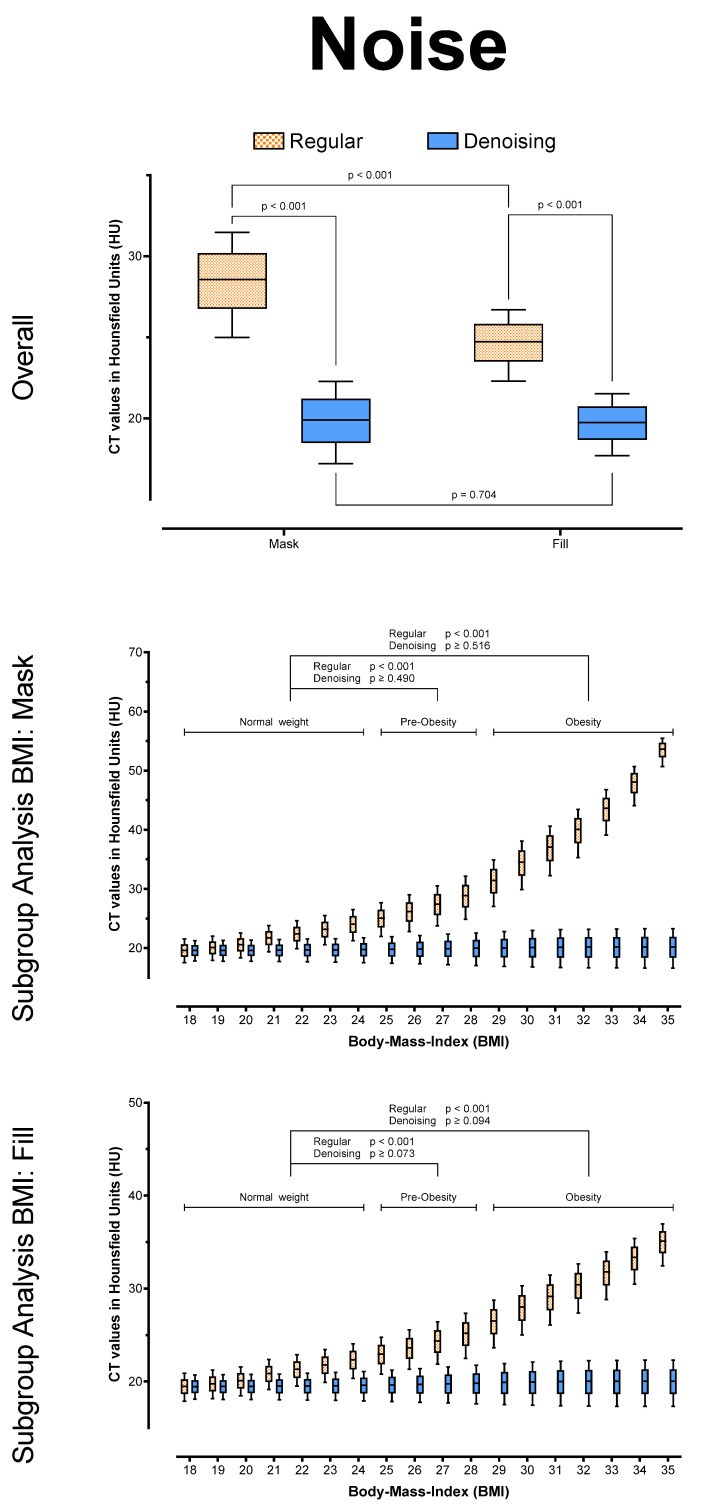
Noise (SD of HU), overall and posthoc subgroup analysis for patient BMI.

**Figure 5:**
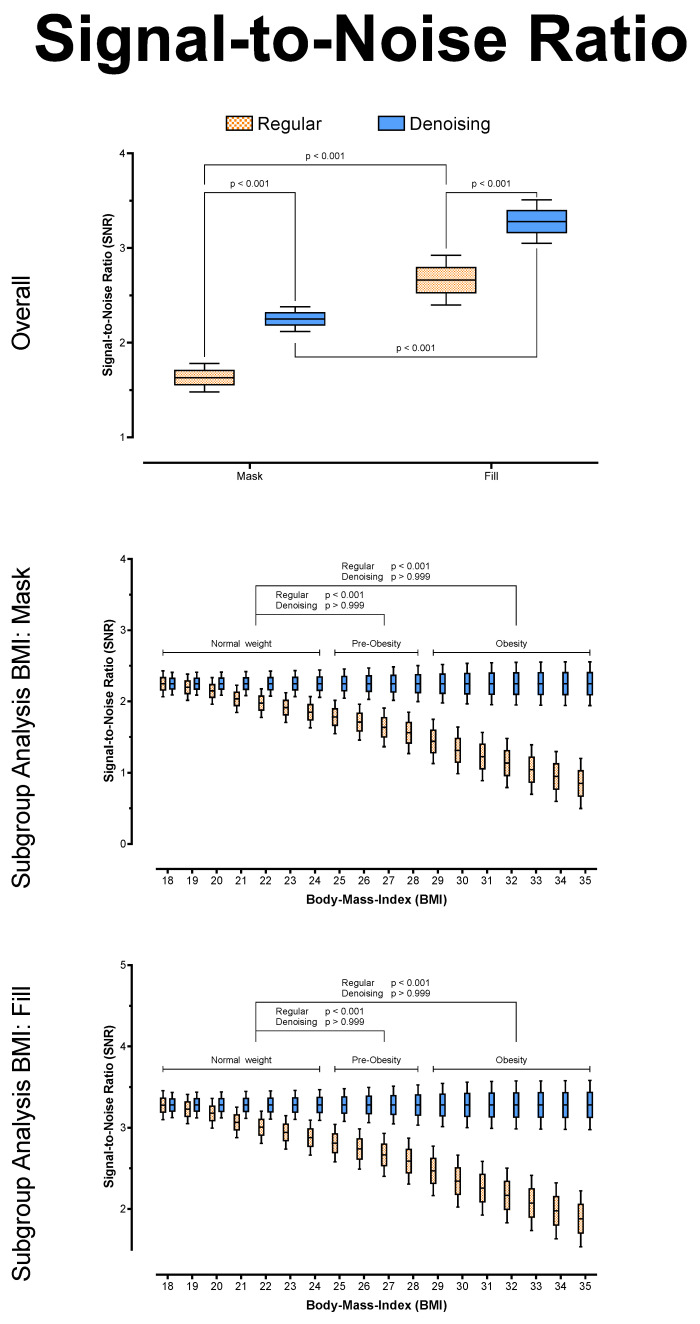
Signal-to-Noise Ratio (HU/SD), overall and posthoc subgroup analysis for patient BMI.

Overall, there were no significant differences between regular and denoised CT numbers in HU (*p* > 0.999) for the mask and the fill run, respectively. However, for both series, the noise was significantly lower in the denoised datasets (*p* < 0.001), and SNR was significantly higher in the denoised datasets (*p* < 0.001), regardless of BMI. In addition, the algorithm reduced noise to equivalent levels in both series (*p* = 0.704) and across all BMI values (Mask: *p* ≥ 0.073; Fill: *p* ≥ 0.490), leading to equally stable SNR values in the denoising datasets (each *p* ≥ 0.999). See Table 2 and Figure 3, Figure 4 and Figure 5 for further details.

The multiple linear regression model was able to predict signal-to-noise ratio (F (3; 5996) = 6616; *p* < 0.001) and showed a high goodness-of-fit (adjusted R2 = 0.77, Sy.x = 0.19). All variables were identified as significant contributors towards SNR (each *p* < 0.001). The variables “BMI” (B = −0.0286) and “Radiation Exposure” (B = −0.005) were associated with SNR-deterioration. Only the variable “Denoising” had a positive impact on SNR, with a mean SNR increase of B = 0.6191. See Table 3 for further regression metrics.

### 3.3. Diagnostic Confidence

Overall, diagnostic confidence was rated good (4 (3–5)) for the regular datasets and excellent (5 (4–5)) for the denoised datasets. Spearman correlation showed good agreement in both datasets (regular: r ≥ 0.859, *p* < 0.001; denoising: r ≥ 0.795, *p* < 0.001). See Table 4 for further details.

**Figure 6:**
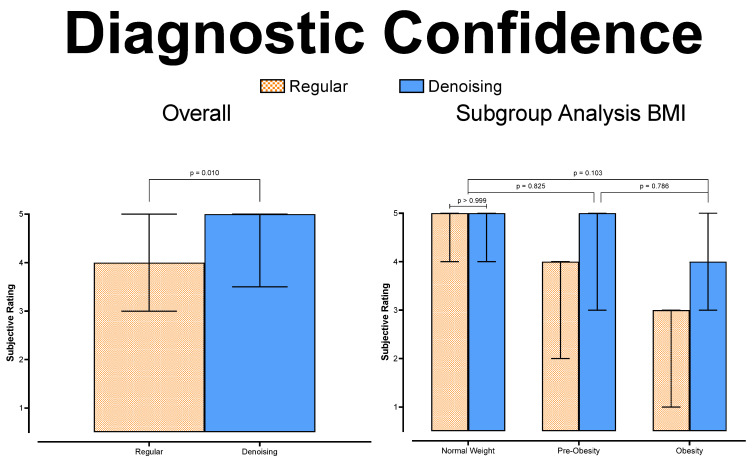
Diagnostic confidence.

In pairwise comparisons, the denoised datasets received overall significantly higher diagnostic confidence grades (F(1; 1) = 4053; *p* = 0.010). In subgroup analysis, diagnostic confidence deteriorated significantly for higher patient BMI (each *p* < 0.001) in the regular datasets. Although there was also a measurable drop in diagnostic confidence in the subgroup analysis of the denoised datasets, this was not statistically significant (*p* ≥ 0.103). Figure 6 visualizes diagnostic confidence scores and pairwise comparisons.

**Figure 7:**
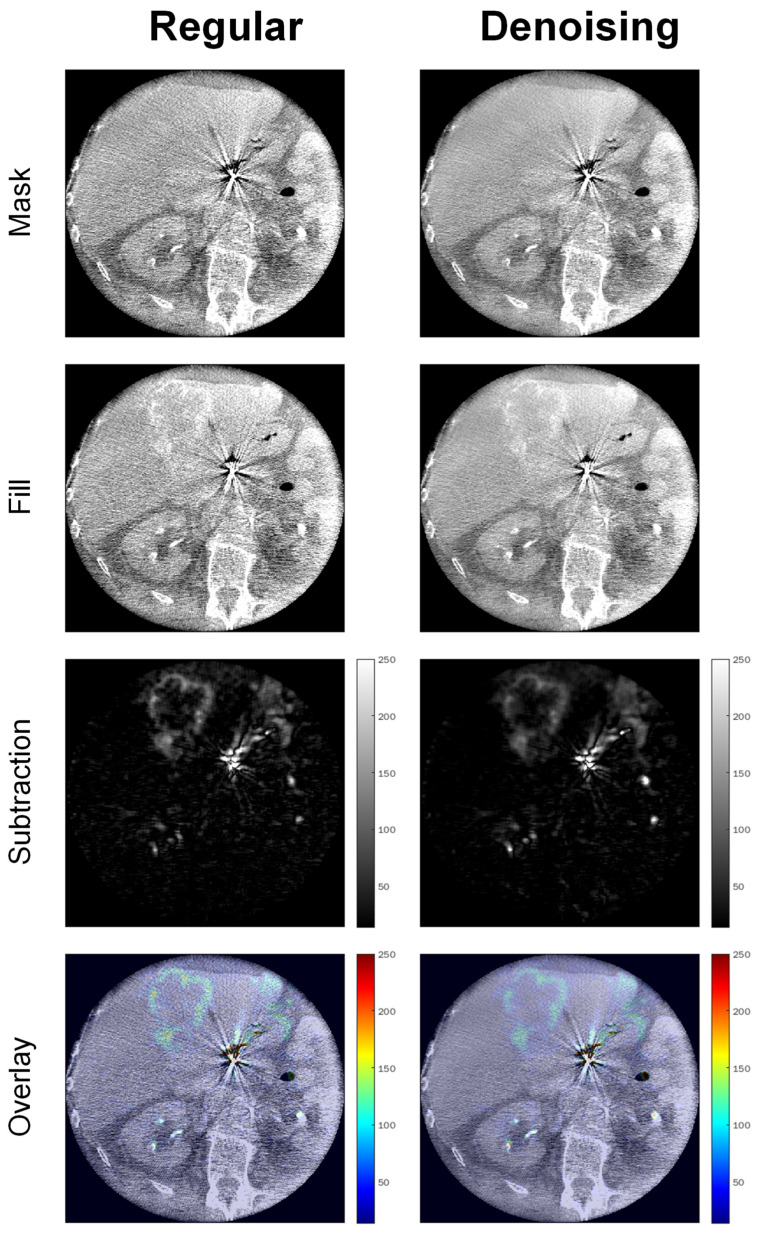
Example images of a 68-year-old adipose male patient (BMI = 30) undergoing SIRT for hepatic uveal melanoma metastases.

Figure 7 shows example images of a 68-year-old adipose male patient (BMI = 30) undergoing SIRT for hepatic uveal melanoma metastases, illustrating significantly enhanced image quality by noise reduction and preserved localizability of the interventional target in the intensity overlay map.

## 4. Discussion

Liver-directed interventional radiology is crucial to modern comprehensive oncology. It may help severely reduce organ tumor burden and thus potentially re-allow curative resection in otherwise non-treatable patients. Multiple studies have pointed out the additional benefit of C-arm cone-beam CT to image-guided procedures via improved visualization. Although modern interventional radiology suites usually include automatic radiation dose adaption to guarantee stable signal yield, image quality deterioration in patients with high BMI can still be challenging. This study examined the impact of a novel AI postprocessing denoising algorithm on interventional cone-beam CT image quality, especially regarding patient BMI.

As expected, we measured rising radiation exposure for rising patient BMI, undoubtedly due to our interventional suite’s automatic dose adaption feature. Although such features aim to stabilize signal yield, we still measured significantly higher image noise in the cone-beam CT of the pre-obesity and obesity subgroups compared to the normal weight subgroup. This result is in line with that of previous publications. Buckley et al. pointed out that the only viable option to improve signal yield in obese patients was to increase the radiation dose [24]. Nonetheless, Fursevich et al. described the significantly higher image noise in the CT of bariatric patients as particularly challenging [25]. In our study, the denoising algorithm could produce stable image noise and signal-to-noise ratio levels across all BMI subgroups. Other studies have described the potential benefit of AI denoising algorithms on CT in obese patients. For example, Tamura et al. described significantly reduced image noise when applying a denoising solution on abdominal CT of obese patients, effectively enabling thin-slice imaging without sacrificing image quality [26]. However, their study focused on conventional CT of obese patients only, so they did not perform BMI subgroup analyses to evaluate the stability of the denoising itself. Zhong et al. proposed a denoising algorithm for cone-beam breast CT capable of reducing noise by 60% [27]. Conveying our results to percentage reduction, we achieved a mean overall noise reduction of 26%, ranging from 11% in the normal-weight group to 41% in the obesity group. Nevertheless, it is worth pointing out that breast imaging typically employs lower kilovolt spectra than interventional liver cone-beam CT, resulting in a higher Poisson noise prevalence per se [28]. Algorithms trained to remove Poisson noise may therefore inherently produce higher results in such setups. In our study, diagnostic confidence was significantly higher in the denoised datasets than in the regular datasets. Prior studies have investigated diagnostic confidence when using AI denoising. Kolb et al. reported lower overall confidence in denoised datasets but stable diagnosis-specific results in their study regarding abdominal CT in patients with suspected appendicitis [29]. However, their results did not encompass patient BMI, so no comparative conclusions could be drawn. Still, it is necessary to reiterate that there also was a measurable drop in diagnostic confidence ratings of the denoised datasets for rising BMI in our study. Although this drop was not statistically significant, our readers pointed out that this might be due to unfamiliar appearances in the denoising datasets, especially in the less intense appearing PBV overlay maps. Nevertheless, we could not measure HU distortions in objective image quality analysis. Therefore, we hypothesize that higher noise in the regular datasets might render the vascular input function calculations more imprecise than in the post-processed datasets. Additionally, our senior radiologist pointed out that the overlays are primarily used as localizers for the interventional target instead of for blood flow quantification. Still, several prior studies have pointed out unfamiliar appearances when using AI denoising. Shin et al. described the loss of spatial information and left-shifted noise-power-spectra in denoised images, especially for lower radiation doses [30]. McCollough et al. reiterated this issue, especially pointing out significant blurring effects [31]. Although our study was not focused on radiation dose reduction, imaging in obese patients may produce similar signal yield insufficiencies [32]. Therefore, we advise cautious optimism when handling novel AI solutions and properly evaluating patient BMI distribution performance stability. Nonetheless, the investigated algorithm not only significantly enhanced image quality and diagnostic confidence of interventional cone-beam CT compared to regular datasets in our study, but it also enabled stable image quality and diagnostic confidence levels across all BMI subgroups. Therefore, interventional radiology may benefit from AI denoising, especially regarding higher patient BMI.

This study has several limitations. First, this was a retrospective study with 100 patients. Although an a priori power analysis confirmed the validity of our experiments in this setup, a prospective follow-up study should explore the impact of AI denoising on angiographic decision-making. Second, we used offline reconstruction. The actual feasibility of implementing AI denoising into clinical workflows was not explored. Third, this study focused on image quality metrics only. As our readers pointed out, denoising produced unfamiliar appearances in some cases and reduced the intensity of the overlay maps. Although this might have been due to misinterpretations in the regular datasets due to higher image noise, quantitative follow-up studies are merited to investigate the actual impact of denoising on time-intensity curves. Last, this study used cone-beam CT examinations from a single interventional suite by one vendor. The generalizability of our results beyond this setup may therefore be limited.

## 5. Conclusions

AI denoising can significantly enhance image quality in interventional cone-beam CT and effectively mitigate diagnostic confidence deterioration for rising patient BMI.

## Figures and Tables

**Table 1:** Patient data.

		Female			Male			Overall
		SIRT	TACE	Overall	SIRT	TACE	Overall	
Number (n)	Overall	13	12	25	37	38	75	100
	HCC	4	9	13	12	35	47	60
	mUM	4	1	5	12		12	17
	CCC	1		1	6	1	7	8
	CRC	2	1	3	2		2	5
	NET	2	1	3	5	2	7	10
Age (y)	Overall	61 ± 15	69 ± 9	65 ± 13	69 ± 12	69 ± 9	69 ± 11	68 ± 11
	HCC	61 ± 17	70 ± 9	67 ± 12	70 ± 13	69 ± 9	69 ± 10	69 ± 11
	mUM	60 ± 9	73	62 ± 10	74 ± 10		74 ± 10	71 ± 11
	CCC	40		40	67 ± 12	64	67 ± 11	63 ± 14
	CRC	61 ± 30	62	61 ± 22	72 ± 8		72 ± 8	65 ± 17
	NET	74 ± 3	61	70 ± 8	57 ± 12	69 ± 6	61 ± 11	63 ± 11
Heigth (cm)	Overall	165 ± 6	161 ± 8	163 ± 7	173 ± 8	173 ± 8	173 ± 8	171 ± 9
	HCC	168 ± 4	162 ± 9	164 ± 8	171 ± 6	173 ± 8	172 ± 7	171 ± 8
	mUM	164 ± 7	157	162 ± 6	173 ± 12		173 ± 12	170 ± 12
	CCC	160		160	174 ± 6	172	174 ± 5	172 ± 7
	CRC	172 ± 1	162	169 ± 6	177 ± 2		177 ± 2	172 ± 6
	NET	159 ± 1	154	157 ± 3	176 ± 4	172 ± 11	175 ± 6	170 ± 10
Weight (kg)	Overall	72 ± 9	70 ± 10	71 ± 9	80 ± 12	79 ± 14	79 ± 13	77 ± 12
	HCC	71 ± 11	69 ± 11	70 ± 11	78 ± 9	79 ± 14	78 ± 13	77 ± 13
	mUM	73 ± 12	69	72 ± 10	81 ± 11		81 ± 11	79 ± 12
	CCC	69		69	83 ± 14	86	84 ± 13	82 ± 13
	CRC	79 ± 4	73	77 ± 4	98 ± 13		98 ± 13	85 ± 14
	NET	70 ± 4	78	72 ± 6	70 ± 11	85 ± 9	74 ± 12	74 ± 10
BMI (kg/m^2^)	Overall	26 ± 3	27 ± 4	27 ± 3	27 ± 4	26 ± 4	27 ± 4	27 ± 4
	HCC	25 ± 3	26 ± 4	26 ± 4	27 ± 3	26 ± 4	26 ± 4	26 ± 4
	mUM	27 ± 3	28	27 ± 3	27 ± 3		27 ± 3	27 ± 3
	CCC	27		27	28 ± 4	29	28 ± 3	28 ± 3
	CRC	27 ± 1	28	27 ± 1	32 ± 5		32 ± 5	29 ± 4
	NET	28 ± 2	33	29 ± 4	22 ± 3	29 ± 1	24 ± 4	26 ± 4

SIRT = selective internal radiation therapy; TACE = transarterial chemoembolization; HCC = hepatocellular carcinoma; mUM = metastasized uveal melanoma; CCC = cholangiocellular carcinoma; CRC = colorectal carcinoma; NET = neuroendocrine tumor.

**Table 2:** Objective image quality analysis.

		Mask			Fill		
	BMI-Group	Regular	Denoising	*p* (Two-Sided, Adjusted)	Regular	Denoising	*p* (Two-Sided, Adjusted)
HU	Overall	44.86 ± 5.79	44.77 ± 5.06	>0.999	64.92 ± 7.5	64.79 ± 6.55	>0.999
	Normal Weight	44.29 ± 4.35	44.27 ± 3.80	>0.999	64.1 ± 5.63	64.07 ± 4.92	>0.999
	Pre-Obesity	44.83 ± 5.62	44.74 ± 4.92	>0.999	64.87 ± 7.28	64.75 ± 6.36	>0.999
	Obesity	45.39 ± 6.95	45.23 ± 6.08	>0.999	65.69 ± 9.00	65.46 ± 7.86	>0.999
Noise	Overall	28.45 ± 6.45	19.84 ± 1.55	<0.001	24.65 ± 3.35	19.70 ± 1.17	<0.001
	Normal Weight	22.48 ± 1.96	19.65 ± 1.17	<0.001	21.34 ± 1.34	19.51 ± 0.88	<0.001
	Pre-Obesity	26.9 ± 2.42	19.83 ± 1.50	<0.001	24.03 ± 1.56	19.69 ± 1.13	<0.001
	Obesity	35.74 ± 5.94	20.02 ± 1.85	<0.001	28.38 ± 2.72	19.89 ± 1.39	<0.001
SNR	Overall	1.63 ± 0.30	2.25 ± 0.08	<0.001	2.66 ± 0.33	3.28 ± 0.14	<0.001
	Normal Weight	1.97 ± 0.14	2.25 ± 0.06	<0.001	3.00 ± 0.17	3.28 ± 0.10	<0.001
	Pre-Obesity	1.66 ± 0.12	2.25 ± 0.08	<0.001	2.69 ± 0.18	3.28 ± 0.14	<0.001
	Obesity	1.29 ± 0.20	2.25 ± 0.10	<0.001	2.32 ± 0.25	3.28 ± 0.17	<0.001

HU = CT numbers in Hounsfield Units, Noise = standard deviation of CT numbers (SD of HU), SNR = Signal-to-Noise Ratio; *p* = significance level.

**Table 3:** Objective image quality analysis.

Variable	B	SE	95% CI (Asymptotic)	|t|	*p*
Intercept	2.813	0.0152	2.783 to 2.843	185.60	<0.001
BMI	−0.0286	0.0068	−0.0419 to −0.0153	4.21	<0.001
Radiation Exposure	−0.0053	0.0002	−0.0058 to −0.0049	23.87	<0.001
Denoising	0.6191	0.0048	0.6096 to 0.6286	127.90	<0.001

BMI = Body-Mass-Index in kg/m^2^; Radiation Exposure = dose area product in mGy*cm^2^; B = regression estimate; SE = standard error; 95% CI = 95% confidence interval; |t| = absolute value of t statistics; *p* = significance level.

**Table 4:** Diagnostic confidence.

		Pooled	Rater 1	Rater 2	r	*p* (Two-Sided, Adjusted)
Regular	Overall	4 (3–5)	4 (3–5)	4 (3–5)	0.913	<0.001
	Normal Weight	5 (4–5)	5 (4–5)	5 (3–5)	0.951	<0.001
	Pre-Obesity	4 (2–4)	4 (3–4)	4 (3–5)	0.859	<0.001
	Obesity	3 (1–3)	3 (1–3)	3 (1–3)	0.926	<0.001
Denoising	Overall	5 (4–5)	5 (4–5)	5 (4–5)	0.834	<0.001
	Normal Weight	5 (4–5)	5 (4–5)	5 (4–5)	0.912	<0.001
	Pre-Obesity	5 (3–5)	5 (3–5)	5 (3–5)	0.925	<0.001
	Obesity	4 (3–5)	4 (4–5)	4 (3–5)	0.795	<0.001

r = Spearman correlation coefficient, *p* = significance level.

## Data Availability

Data is contained within the article.
